# Targeting dormant phenotype acquired mycobacteria using natural products by exploring its important targets: *In vitro* and *in silico* studies

**DOI:** 10.3389/fcimb.2023.1111997

**Published:** 2023-03-24

**Authors:** Shweta Sharma, Rupesh Chikhale, Nivedita Shinde, A. M. Khan, Vivek Kumar Gupta

**Affiliations:** ^1^ Department of Biochemistry, ICMR-National JALMA Institute for Leprosy and Other Mycobacterial Diseases, Agra, India; ^2^ Division of Pharmacy and Optometry, University of Manchester, Manchester, United Kingdom; ^3^ Department of Pharmaceutical & Biological Chemistry, School of Pharmacy, University College London, London, United Kingdom; ^4^ Division of Clinical Trials and Implementation Research, ICMR-National JALMA Institute for Leprosy and Other Mycobacterial Diseases, Agra, India

**Keywords:** tuberculosis, mycobacterium, dormancy, drug targets, phytomolecules, LTBI (latent TB infection), homology modeling, molecular docking

## Abstract

The dormant phenotype of *Mycobacterium tuberculosis* that develops during infection poses a major challenge in disease treatment, since these bacilli show tolerance to front-line drugs. An *in vitro* hypoxia dormancy model was established, which produced phenotypically dormant *Mycobacterium smegmatis* after prolonged incubation under conditions of low oxygen, low pH, and nutrient limitation. Bacilli in this model displayed the classical dormancy characters, including loss of acid fastness, altered morphology, and, most importantly, tolerance to front-line drugs. The dormant form of *M. smegmatis* was treated with drugs and phytomolecules. Three phytomolecules exhibited activity against dormant bacilli, as shown by lack of regrowth in solid and liquid media. Further investigation of dormancy-active hits was carried out using *in silico* approaches to understand the druggable targets of these phytomolecules in dormant bacilli. For this study, molecular docking, molecular dynamics simulations (MDS), and molecular mechanics-generalized born solvent accessibility **(**MM-GBSA)-based binding energy (ΔG_bind_) calculations were performed. Five different targets, namely, isocitrate lyase (ICL), GMP synthase, LuxR, DosR, and serine/threonine protein kinase (STPK), from *M. smegmatis* and *M. tuberculosis* were studied in details. DosR and STPK were found to be the common targets in both the species that were more prone to the phytomolecules. The standard DosR inhibitor, HC104A, showed a lower dock score and binding energy of −4.27 and −34.50 kcal/mol, respectively, compared to the natural products under study. The phytomolecule, icariin, showed better docking score (dock score = −5.92 kcal/mol with and binding energy ΔG_bind_= −52.96 kcal/mol) with DosR compared to known DosR inhibitor, HC104A (dock score = −4.27 kcal/mol and binding energy ΔG_bind_ = −34.50 kcal/mol). Similarly, the known STPK inhibitor MRCT67127 showed a lower dock score and binding energy of −4.25 and −29.43 kcal/mol, respectively, compared to the phytomolecule, icariin (dock score = −5.74 kcal/mol and ΔG_bind_= −43.41 kcal/mol). These compounds might ultimately lead to new therapeutics or may be useful as adjuvants to the first-line drugs to reduce the lengthy anti-TB therapy in the future.

## Introduction

Tuberculosis (TB) is one of the main causes of death by an infectious disease worldwide. A large number of individuals latently infected with *Mycobacterium tuberculosis* (*Mtb*) delay the efforts to eliminate this disease (Ai et al., 2016). The dormant form of bacilli in latent TB infection (LTBI) also serves as a reservoir for the reactivation and transmission of the disease. The lifetime risk of reactivation of bacilli in LTBI is estimated to be 5%–10%, and this is 10–100 times more in HIV patients (Sterling et al., 2020). Targeting the dormant form of *Mtb* in LTBI is a big challenge for current chemotherapy (Dutta and Karakousis, 2014). The dormant state of *Mtb* is characterized by the presence of non-dividing or slowly replicating bacilli with low metabolic state, and such bacilli are also phenotypically resistant to standard anti-tuberculosis (anti-TB) agents (Gupta et al., 2017). Complete cure of TB requires the eradication of both replicating and dormant forms of *Mtb*. Most of the standard anti-TB agents are more than 45 years old and are mostly active against the replicating form of bacilli. A prolonged therapy is required due to dormancy-induced drug tolerance developed by *Mtb* (Evangelopoulos et al., 2015). The differences in altered physiology of the dormant bacilli from replicating bacilli can serve as therapeutic drug targets for the development of effective therapy against this disease.

The search for antimycobacterial compounds from natural sources is a historically authenticated approach. Plant molecules are the major component of traditional medicine system, which is well known to possess a wide range of biological activities, such as antioxidative, anti‐inflammatory, antimicrobial, and anticancer effects (Cowan, 1999).

In this study, three plant molecules, namely, ursolic acid (UA), betulinic acid (BA), and icariin (IC), were tested against the dormant form of *Mycobacterium smegmatis*, which serves as a surrogate model strain for the pathogenic *Mtb* (Chaturvedi et al., 2007). An *in vitro* hypoxia dormancy model developed by Wayne and Hayes (1996) was used in this study, which produced dormant bacilli after prolonged incubation under conditions of low oxygen, low pH, and nutrient limitation (Wayne and Sramek, 1994). Bacilli in this model exhibited the typical dormancy traits, including loss of acid fastness, altered morphology, and, most importantly, tolerance to front-line anti-TB drugs.

Further investigation of dormancy-active hits was carried out using *in silico* approaches to understand the druggable targets of these phytomolecules in dormant bacilli, which may ultimately lead to new therapeutics that may be able to reduce the lengthy anti-TB therapy.

## Materials and methods

### Maintenance of *M. smegmatis*

*Mycobacterium smegmatis* strain (MTCC 994) was procured from Microbial Type Culture Collection (MTCC), Institute of Microbial Technology, (IMTECH), Chandigarh, India. *M. smegmatis* was maintained at pH 7 and temperature at 37°C in Middlebrook 7H9 (Difco) liquid culture medium supplemented with 10% oleic albumin dextrose catalase (OADC) (BD) and 0.1% Tween-80 (Merck). Nutrient agar (Hi-Media) was used as solid culture medium for the routine maintenance of culture.

### Drugs and plant compounds

All antibiotics and compounds were purchased from Sigma-Aldrich Chemicals (St. Louis, MO, USA). The standard first-line antibiotics used included rifampicin (RIF) and isoniazid (INH). INH and metronidazole were each prepared in water to stock concentrations of 10 and 50 mg/ml, respectively, while RIF was dissolved in methanol (stock concentration, 10 mg/ml). The three phytomolecules were dissolved in dimethyl sulfoxide (DMSO) (Merck) to a stock concentration of 10 mg/ml. The final working concentration ranges for each antibiotic were prepared by twofold serial dilutions with appropriate solvents.

### Generation of dormant bacilli

In order to generate *in vitro* dormancy, a hypoxic model developed by Wayne and Hayes (1996) was used for this study. Broth culture of *M. smegmatis* was taken in tubes (with tight-fitting rubber caps), which were then sealed and centrifuged at 6,000 rpm for 10 min at 10°C. The pellet was washed twice with phosphate-buffered saline (PBS) and then dissolved in PBS with Tween-80 (0.01% v/v). The culture was diluted in nutrient-deficient Sauton’s medium (Mulyukin et al., 2010; Nikitushkin et al., 2016) (**Supplementary Table S1**) containing pH 6.0 to reach a dilution of 1:10. (1.0 McFarland suspension). The culture was divided into two vacutainer tubes (5.0 ml) (Becton Dickinson) (Rathor et al., 2016; Imperiale et al., 2017), which were tightly screwed and sealed with parafilm. The methylene blue was added to the final concentration of 1.5 µg/ml to one of the tubes as an indicator of hypoxia (oxygen depletion). The optimal headspace ratio of air volume to liquid was maintained in the tubes throughout the experiment. The tubes were incubated at low stirring at 37°C to maintain a hypoxic environment. Regular observations were made for the reduction in methylene blue, and colony-forming units (CFU) counting was performed.

### Ziehl–Neelsen staining

A thin bacterial smear was prepared from a hypoxic culture tube to perform Ziehl–Neelsen (Z–N) staining using carbol fuchsin (Van Deun et al., 2008). To decolorize the background, sulfuric acid (20% v/v) was used. Counterstaining was done using methylene blue, and slides were observed under a 100× oil immersion microscope.

### Electron microscopy

Scanning electron microscopy (SEM) of *M*. *smegmatis* culture was performed to observe their morphology under stress environment. Cultures from above experiments were taken for this study. The cells were fixed overnight at 4°C in a mixture of 4% (v/v) paraformaldehyde (Sigma-Aldrich) and 2.5% (v/v) glutaraldehyde (Sigma-Aldrich) in 0.1 M phosphate buffer (pH 7.4). The sample suspension was then centrifuged, the supernatant was discarded, and the pellet was washed with 0.1 M phosphate buffer twice; the specimens were then fixed with 1% (w/v) osmium tetraoxide (Sigma-Aldrich) in 0.1 M phosphate buffer for 1 h. The cells were then dehydrated in ascending concentration using molecular grade ethanol (Merck) from 50%, 70%, and 100% (10 min at room temperature). The cells were transferred on a glass coverslip and air dried. All samples were coated with gold using a sputter coater (Quorm, SC7620) and examined using a scanning electron microscope (THERMO FEISEM, Volumescope) (Kim et al., 2017; Pawar et al., 2020). SEM was performed at the Advanced Technology Platform Centre (ATPC) facility, RCB, Faridabad, India.

### Hypoxic resazurin reduction assay for screening of compounds against dormant bacilli

HyRRA was performed with slight modification as described by Taneja and Tyagi (2007). Briefly, vacutainer tubes (5.0 ml) were filled, using a syringe and needle (26½), with nutrient-deficient Sauton’s medium. *Mycobacterium smegmatis* (from hypoxic culture of the above experiment) was inoculated in vacutainer tubes to a turbidity equivalent to 1.0 McFarland turbidity suspension.

The tubes were kept static at 37°C to allow for self-generation of hypoxia in the cultures (as described above). Fading/decolorization of the methylene blue dye (at final concentration of 1.5 µg/ml) was used to determine hypoxia. Various test compounds/drugs were subsequently injected at different concentrations by a needle without opening the tubes and further incubated for 48 h at 37°C under static conditions. Metronidazole (actively works on anaerobically grown microorganisms) and isoniazid (primarily acting on dividing cells) were used as positive control. Resazurin dye (0.02%) was added in each vacutainer tube. A reduction in dye color was noted after 24 h of incubation at 37°C. Minimum inhibitory concentrations (MICs) were determined for the test drugs in the same manner as for aerobic resazurin microtiter plate assay (REMA) (Martin et al., 2003).

*Statistical analysis*. GraphPad Prism 5 software (Graph Pad Software, San Diego, CA) was used for analysis of data.

### Molecular docking

#### Homology modeling

The protein crystal structures for the *M. smegmatis* isocitrate lyase (ICL), GMP synthase, LuxR, DosR, and serine/threonine protein kinase (STPK) are not available. These structures were modeled by homology modeling using tools like Swiss-Model (https://swissmodel.expasy.org/) (Waterhouse et al., 2018) and AlfaFold (https://alphafold.ebi.ac.uk/) (Jumper et al., 2021; Varadi et al., 2022). The modeled structures were validated for their structural quality by means of Ramachandran Plot, Qmean, and MolProbity scores (Benkert et al., 2009, 2011)

#### Protein preparation

The crystal structures for DNA-binding transcriptional activator DosR/DevR (PDB: 3C3W) (Wisedchaisri et al., 2008) and intracellular STPK domain of *Mtb* PknB (PDB: 1MRU) (Young et al., 2003) from *Mtb* were obtained from the Protein Data Bank (PDB) (https://www.rcsb.org/). These were directly imported into the protein preparation wizard of Schrödinger Maestro version 2018-1 (Schrödinger, 2018). These structures were analyzed for any inconsistencies like breaks or missing residues and was prepared by adding hydrogen, treating the metal, deleting water molecules beyond 5 Å, correcting for protonation states, and assigning partial charges by using the OPLS-2005 force field. Both of these crystal structures are apo proteins; the binding site was determined using the SiteMap tool, and the top-ranking site was selected to prepare a grid using a protein–ligand docking grid generation tool in Maestro.

#### Ligand preparation

The 2D structure of ligands, namely, ursolic acid (UA), betulinic acid (BA), and icariin (IC), were downloaded from PubChem compound database (https://pubchem.ncbi.nlm.nih.gov) in SDF format and uploaded to the work space in Glide. The ligands HC104A and MRCT67127 were used as standard ligands. The LigPrep (Schrödinger, 2018) module in Maestro was used to generate three-dimensional conformations of these ligands with a higher limit of 200 conformations for each structure; all the conformations generated were further used for docking simulations.

### Protein ligand docking

The molecular docking simulations were performed in the Glide module of the Schrödinger suite with the grid file for protein binding site and ligand conformations generated in earlier steps. The docking was performed in the extra precision mode (XP mode) and the poses with high docking score, protein–ligand interactions like hydrogen bond, and π–π interactions were selected. The results of simulations were analyzed using a glide XP visualizer, and the important active site interactions were analyzed along with the scoring functions.

### Molecular dynamics simulations and molecular mechanics-generalized born solvent accessibility analysis

#### System preparation

All the 29 molecular dynamics simulations were calculated on AMBER 18 software package (Lee et al., 2018; Singh et al., 2020). ANTECHAMBR (Wang et al., 2001) was used for ligand preparation and for the determination of the charges on the ligand; further GAFF force field was used for parametrization, and the protein–ligand complexes were prepared in XLEAP (Wang et al., 2001). The protein receptor–ligand complex was solvated separately in truncated octahedron of TIP3P (Price and Brooks, 2004) box with water molecules; a sufficient number of counter ions Na^+^ and Cl^−^ were added to neutralize the simulation system, and 0.1M of ionic strength was achieved (Mark and Nilsson, 2001; Price and Brooks, 2004). To parameterize the amino acids and to model the proteins, FF14SB force field was used (Maier et al., 2015).

#### Unbiased MD simulation

All simulations were performed for 100 ns on Nvidia V100-SXM2-16GB Graphic Processing Unit using the PMEMD.CUDA (Peramo, 2016) module installed on the Computational Shred Facility (CSF), University of Manchester, UK. Simulations were run at 1 atm constant pressure using Monte Carlo barostat and 300 K constant temperature by using Langevin thermostat with a collision frequency of 2 ps^−1^, and the volume exchange was attempted for every 100 fs. An integration step of 2 fs was also used for the simulation of the hydrogen atoms involving bonds and were constrained by using SHAKE algorithm (Andersen, 1983). Long-range electrostatic interactions were computed by using the particle mesh Ewald method, while for short-range interaction, a cutoff of 8 Å was used. Equilibration consisted of rounds of NVT and NPT equilibrations, respectively, for 10 ns in total. CPPTRAJ (Roe and Cheatham, 2013) was used to analyze the interactions over full trajectory after taking configuration at every 4 ps. Root mean square deviation (RMSD), root mean square fluctuation (RMSF), and MM-GBSA binding free energy were determined after analyzing the trajectories.

### MM-GBSA analysis

The MM-GBSA was performed on Amber18 and Amber18 tools. After simulation of the protein–ligand complexes, all the trajectories of 100 ns covering all the 10,000 frames were used for MM-GBSA analysis (Rastelli et al., 2010). All the results in the form of energies were tabulated and reported in kcal/mol.

## Results and discussion

### Evaluation of the *in vitro* dormancy model

In order to mimic the granuloma stress *in vitro* to generate dormancy, Wayne’s hypoxia model was used in this study for the generation of dormant bacilli. The additional stress of nutrient starvation with slightly acidic pH (6.0) were also provided. With the use of this model, we observed bacterial growth in 1.75±0.52 × 10^9^ CFU in vacutainer tubes at day 7, indicative of the replication stage of bacterial culture (**Supplementary Figure S1**). At this stage, the color of methylene blue was not changed, which showed the presence of normal environment in the culture vials. On the 14th day, the color of methylene blue was reduced, indicating a generation of hypoxia, which easily correlated with the decrease in the number of cells corresponding to the stress (Dick et al., 1998). A 100-fold reduction in CFU was observed in the vials at day 14. The growth of the bacteria became static from 21 days onward. The shifting of the environment from aerobic to hypoxic/anaerobic and nutrient limitation may be the cause of the decrease in CFU. Once bacteria adapt to the stress environment, the static condition is maintained for a long time. All the experiments were performed in triplicates.

The generation of dormancy was also validated through the Z-N staining method. The Z-N staining differences in cultures were observed in this study. The aerobic replicating culture showed a darker pink shade, while the hypoxic culture showed a lighter tone of pink (**Supplementary Figure S2**). The dormant cells contain less mycolic acid in their cell wall and thus take up a lower stain as compared to active cells (Deb et al., 2009).

### Electron microscopic studies

**Figure 1:**
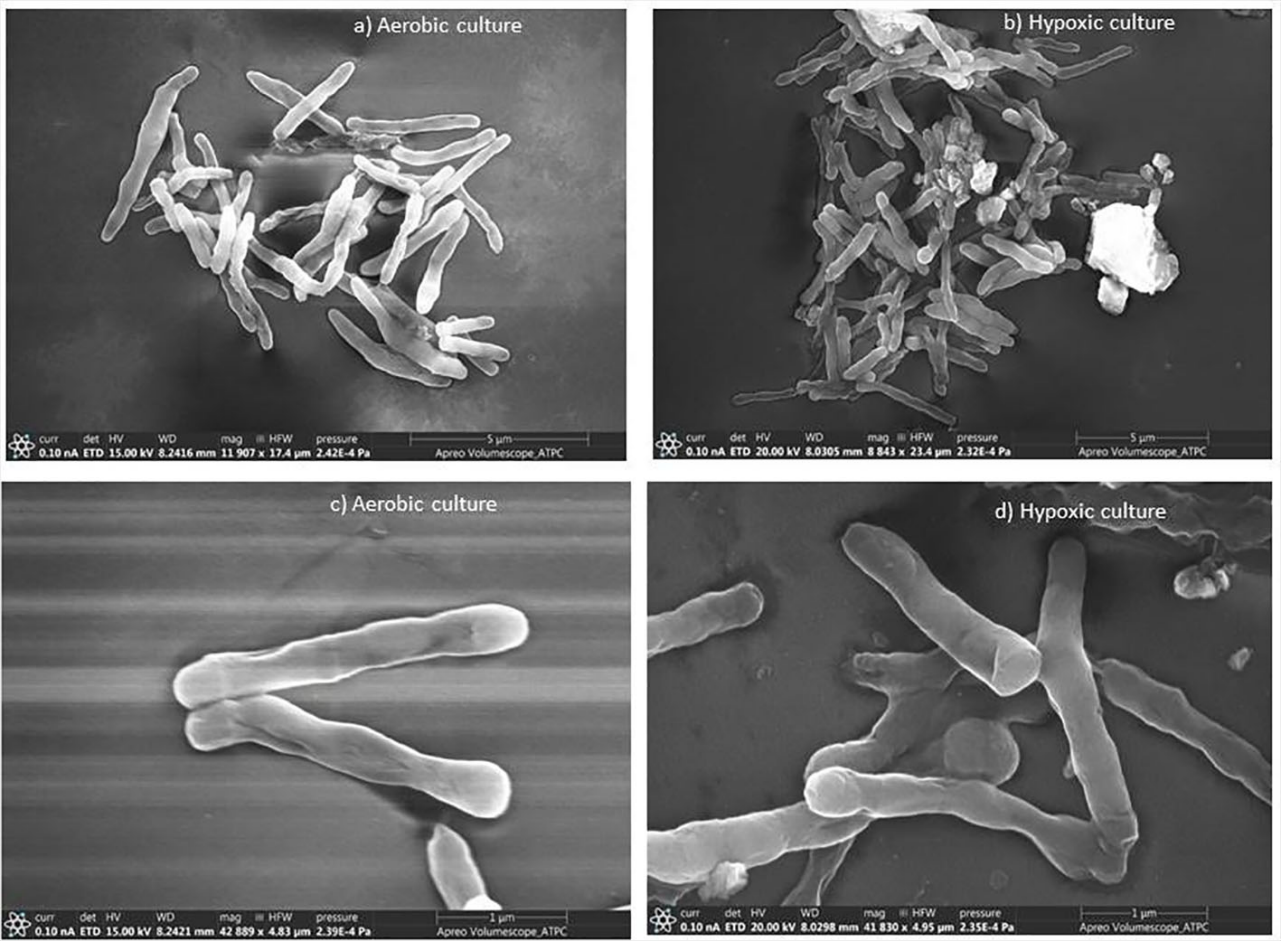
Scanning electron micrographs of *M. smegmatis* under aerobic and hypoxic environment: **(A, C)** aerobic culture and **(B, D)** hypoxic culture.

*Mycobacterium smegmatis* cells from aerobic culture (**Figures 1A, C**) have smooth a surface and well-defined rigid shape. These cells were mostly rod-like proper length with septa or constrictions. In hypoxic culture, slight morphological changes were observed in comparison to the aerobic culture. The culture under hypoxic environment showed apical swelling, and the lengths of most of the bacilli were short (**Figures 1B, D**). Trutneva et al. (2020) observed altered morphology (small and ovoid appearance) of dormant cells of *Mtb* after 4.5 months of incubation in comparison to multiplying bacteria (typical rod-shaped appearance).

### Evaluation of anti-TB activity of phytomolecules against dormant bacilli

The aim of our work was to determine the potent antibacterial activity of UA, BA, and IC, and their possible use as new agent against dormant bacterial population.

After the generation and validation of *in vitro* dormancy model, the dormant cells were used to screen the activities of phytomolecules to understand their efficiency. Three different concentrations (500, 250, and 125 µg/ml) of phytomolecules were used for the treatment of dormant cells (**Table 1**). At minimum inhibitory concentration (MIC) of phytomolecules, cultures remained blue post-resazurin (**Supplementary Figure S3**) addition and thereby confirmed their bactericidal activity against dormant *M. smegmatis*, and no growth was observed on solid and liquid media. Our results revealed that all the three phytomolecules exhibited significant anti-mycobacterial activity against dormant bacilli. UA, BA, and IC were effectively active against the dormant form of *M. smegmatis* in this study. UA, BA, and IC also exhibited activity against the replicating form of *M. smegmatis* with MICs of 125, 250, and 500 µg/ml, respectively.

**Table 1:** MIC of each compound against the *in vitro* generated dormant cells of *M. smegmatis*.

Sr. No.	Phytomolecules	MIC (µg/ml)
**1**	Betulinic acid	125
**2**	Icariin	500
**3**	Ursolic acid	125
**4**	Metronidazole (positive control)	50
**5**	Rifampicin (standard drug)	>60
**6**	Isoniazid (standard drug)	50

Hence, our results agree with those from a previous study, which noticed that UA, BA, and IC were active against replicating form of *Mtb* (Jiménez-Arellanes et al., 2013; Lone et al., 2017).

Rifampicin and isoniazid were used as standard anti-TB drugs, while metronidazole was the positive control in the *in vitro* experiment. The dormant bacilli showed phenotypic drug resistance against standard drugs rifampicin and isoniazid. In the presence of metronidazole at 50 µg/ml, the blue color of resazurin did not change, which confirmed their activity under hypoxic environment (Wayne and Sramek, 1994; Murugasu-Oei and Dick, 2000).

### *In silico* study

In order to understand the molecular mechanism of inhibition taking place inside the dormant cell, *in silico* studies were also undertaken against important drug targets in dormant *M. smegmatis* and *Mtb.*

*Mtb* face depletion of oxygen and nutrients in the stress environment of the host’s granuloma and survive in the form of non-replicating dormant cells. Isocitrate lyase (ICL) plays an important role in the survival of latent *Mtb* during chronic stage of infection (McKinney et al., 2000). ICL is a glyoxylate cycle enzyme, which converts isocitrate to succinate and glyoxylate, followed by the addition of acetyl-CoA to glyoxylate to form malate by malate synthase, which help survival of *Mtb* in a carbon-deficient environment. Similarly, the two-component DOS (dormancy survival) regulon enables bacterial adaptation and survival in non-replicating persistence (NRP) stage during dormancy (Dasgupta et al., 2000). The expression of the ~50 gene regulon is coordinated by dormancy survival regulator (DosR), which binds to target sites on DNA and induces transcription of other genes, which help in survival (Sharma and Tyagi, 2016). Fang et al. (2013) detected the vital role of LuxR family regulator Rv0195 in *Mtb* dormancy, which affected the expression of more than 180 genes. Serine/threonine protein kinases (STPKs) play important roles in bacterial cellular processes including cell division, cell wall synthesis, cell metabolism, and dormancy exit (Chawla et al., 2014). Guanosine monophosphate synthase (GMP synthase), encoded by guaA gene (Rv3396c), catalyzes the conversion of xanthosine 5′-monophosphate (XMP) into guanosine 5′-monophosphate (GMP) and is a key enzyme in both purine *de novo* and salvage pathways of guanine nucleotides (Villela et al., 2015).

Considering the important role of the above-mentioned proteins in the dormant stage of the mycobacteria were selected for *in silico* studies. These targets were explored to understand which one of these could potentially be interacting with the ligands under investigation. We started with molecular docking studies to understand the drug–receptor interactions. These docked complexes were further explored by molecular dynamics studies to understand the stability of the complex. The binding energies were calculated by the MMGBSA method. In the docking studies, we docked BA, IC, UA, HC104A (a known DosR inhibitor), and MRCT67127 (a known STPK inhibitor). There are no known crystal structures for the *M. smegmatis* ICL, GMP synthase, LuxR, DosR, and STPK. To overcome this short fall, these proteins were modeled in the SWISS-MODEL using the sequences available on the Uniprot (https://www.uniprot.org/), and these were verified with the structures available in the AlphaFold database (https://alphafold.ebi.ac.uk/). The details of the sequences used for modeling the protein by homology modeling is provided in **Supplementary Table S2**, where the validation parameters like RC-plot, Q-mean scores, and MolProbility scores are provided in more detail. The modeled structures were found to be well within the acceptable ranges for structural quality. This was also verified by comparing the structures available in the AlphaFold database for structural identity. The protein structures were further refined by means of MDS for 100 ns and later used in the molecular docking and MDS studies. These refined protein structures were explored for possible binding sites by the SiteMap tool, the best site used for the generation of Grid for docking in the Glide. The ligands under study were subjected to conformation generation by LigPrep tool in the Glide software.

The docking of ligands BA, IC, UA, HC104A, and MRCT67127 showed several interactions with *M*. *smegmatis* ICL (**Supplementary Figures 4A–C**). The ligand BA docked in the binding site by forming a hydrogen bond interaction with the Lys189 (BA-O3—NZ-Lys, 2.81 Å) with a dock score of −2.85 kcal/mol. IC formed several interactions with the binding site residues of ICL, Thr347 (IC-O8—OG1-Thr, 2.81 Å), Glu285 (IC-O6—OE2-Glu, 2.79 Å), Asp108 (IC-O7—OD2-Glu, 2.61 Å), and Ala233 (IC-O15—O-Ala, 3.16 Å) with a dock score of −3.54 kcal/mol. The ligand UA docked inside the binding site with one hydrogen bond with the residues, Lys189 (UA-O3—NZ-Lys, 3.16 Å) and Asn313 (UA-O1—ND2-Asn, 2.77 Å) with a dock score of −5.95 kcal/mol (**Supplementary Table S3**), respectively. The docking experiment with *M. smegmatis* ICL shows best interaction with the ligand IC. The docking of ligands BA, IC, UA, HC104A, and MRCT67127 showed several interactions with *M. smegmatis* GMP synthase (**Supplementary Figures S4D–F**). To perform in-depth investigation on the binding of BA, IC, and UA with the GMP, we performed MDS for 100 ns on each of the respective complex and calculated the binding energies (**Supplementary Table S3**). These bound ligands showed high ligand RMSDs during the MDS with BA fluctuating from 3 to 15 Å and then lowered down to approximately 4 Å (**Supplementary Figures S5A, D**). IC had a stable initial phase of MDS for approximately 50 ns after which a steep rise to 16 Å was observed, which is probably due to the ligand’s conformational change from its initial docked pose (**Supplementary Figures S5B, E**); its higher binding energy of (ΔG_bind_ = −32.90 (4.90) kcal/mol) indicates that the new conformation might be more favorable than the docked pose. UA-ICL complex was also studied by MDS for 100 ns; the ligand RMSD was found to show high fluctuation through the simulation. Initially, the UA RMSD was approximately 4.5 Å; this increased gradually over the period of 80 ns to approximately 10 Å and lowered to approximately 7.5 Å towards the end of the simulation. The UA–ICL protein RMSD also suggested for high protein RMSD to approximately 6 Å, and the protein RMSF showed high fluctuations for the residues between 350 and 375. The overall observations for UA–ICL complex showed week binding interactions, which was also supported by a low binding energy (ΔG_bind_ = −21.09 (3.79) kcal/mol) (**Supplementary Figures 5C, F**). The docked ligand–GMP synthase complex with BA did not show any hydrogen bond interaction. It has a very low dock score of −1.89 kcal/mol. The ligand UA bound to the *M. smegmatis* GMP synthase showed the formation of two hydrogen bonds, His176 (UA-O3—ND1-His, 2.79 Å) and Asp479 (UA-O1—OD1-Asp, 3.12 Å). The dock score for the *M. smegmatis* GMP synthase–UA complex was −2.72 kcal/mol. IC formed several interactions with the binding site residues of GMP synthase, Phe107 (IC-O15—N-Phe, 2.91 Å), Phe107 (IC-O14—N-Phe, 2.86 Å), Ser105 (IC-O14—O-Ser, 2.86 Å), and Asp479 (IC-O8—OD2-Asp, 2.74 Å) with a dock score of −5.29 kcal/mol. The BA, IC, and UA were also studied in more details for their binding modes with the GMP; the MDS calculations were performed, and binding energies were calculated (**Supplementary Figures S6A–F**). The MDS of GMP complexes for 100 ns showed varied results. The ligand BA’s RMSD over the MDS showed large fluctuations with steep rise from 1 to 12 Å over a short period of 20 ns and kept fluctuating for the remaining time of simulation. Ligand IC showed a lower fluctuation during the MDS. It showed a gradual rise from 2 to 5 Å over a period of 45 ns, after which it stabilized for the rest of the MDS. The ligand UA presents large fluctuations for the initial 50 ns, after which it converged approximately 5 Å for rest of the period. The higher binding energies of ΔG_bind_ = -36.39 (5.41) kcal/mol for the IC–GMP complex supported the higher stability of this complex compared to other GMP complexes. The docking of ligands BA, IC, UA, HC104A, and MRCT67127 showed several interactions with *M. smegmatis* LuxR (**Supplementary Figures S4G–I**). The ligand IC was the only one molecule that showed some hydrogen bond interactions with LuxR, the Trp37 (IC-O7—O-Trp, 2.92 Å) with a dock score of −4.41 Kcal/mol. The MDS for BA–LuxR showed an unstable complex with very high fluctuations. The ligand RMSD fluctuated as high as 80 Å, which lowered to 40 Å towards the end of the simulation (**Supplementary Figures S7A, D**). The IC–LuxR complex was found to be most stable complex with ligand RMSD approximately 3 Å throughout the simulation period; this was also supported by a high binding energy of ΔG_bind_ = −45.42 (5.15) kcal/mol. The UA–LuxR complex showed a low ligand RMSD but a high fluctuating protein RMSD; it showed a large variation of 1.5–5 Å through the MDS, suggesting that UA might not be able to stabilize the residues in the binding site. This was also observed in the protein RMSF with comparative higher fluctuations for residues between 80 and 100, and 130 and 150 of LuxR (**Supplementary Figures S7B–F**). These observations for binding of BA, IC, and UA to the *M. smegmatis* target receptors ICL, GMP, and LuxR by *in silico* methods indicated lower or no specificity towards these proteins.

**Figure 2:**
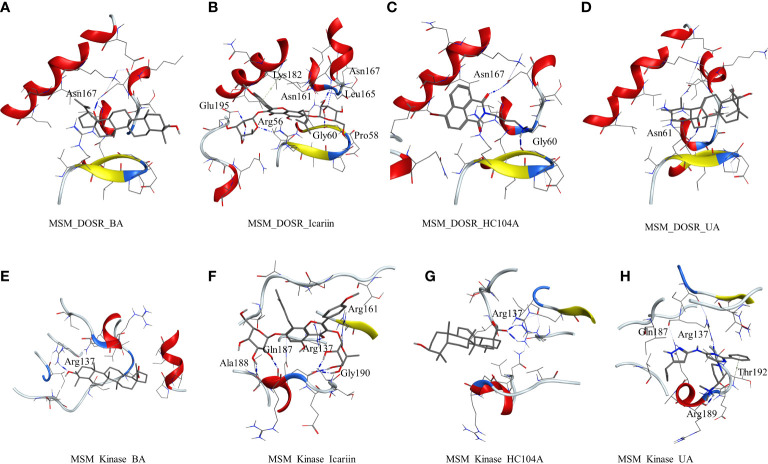
Molecular docking results for *M. smegmatis* dormancy survival regulator (DosR): **(A)** DosR–betulinic acid (BA), **(B)** DosR–icariin (IC), **(C)** DosR–HC104A, and **(D)** DosR–UA. Molecular docking results for *M. smegmatis* serine/threonine-protein kinase (STPK): **(E)** STPK – BA, **(F)** STPK–IC, **(G)** STPK–UA, and **(H)** STPK–MRCT67127.

The other studied target in *M*. *smegmatis* was DosR. The ligands under investigation were docked in the binding site of DosR to understand the ligand–receptor interactions. Compound BA docked in the binding site of the DosR to form a hydrogen bond interaction with Asn167 (BA-O3—ND2-Asn, 2.74 Å) with a dock score of −3.24 kcal/mol (**Figure 2A** and **Table 2**). IC docked in the binding site by interacting with various residues, namely, Arg56 (IC-O12—NH2-Arg, 3.10 Å), Pro58 (IC-O9—O-Pro, Asn61 (IC-O7—ND2-Asn, 3.26 Å), Glu195 (IC-OE2—NH2-Glu, 2.83 Å), and Asn167 (IC-O6—N-Asn, 2.96 Å) with a docking score of −5.92 kcal/mol (**Figure 2B** and **Table 2**). The ligand HC104A was docked in the binding site with few interactions with the surrounding residues, Gly60 (HC104A-O1—O-Gly, 2.84 Å) and Asn167 (HC104A-O1—ND2-Asn, 3.15 Å), and had a dock score of −4.27 kcal/mol (**Figure 2C** and **Table 2**). UA bound to the DosR showed a single hydrogen bond with the residue Leu161 (UA-O2—ND2-Leu, 2.79 Å) with dock score of −3.55 (**Figure 2D** and **Table 2**). The docking results suggest IC as the compound showing better interaction with the *M. smegmatis* DosR receptor as compared to other ligands. The final *M. smegmatis* target studied was STPK, the ligands under investigation were docked in the binding site of STPK to understand the ligand–receptor interactions. Compound BA docked in the binding site of the STPK showed a hydrogen bond interaction with Arg137(BA-O1—ND2-Arg, 2.88 Å) with a dock score of −2.24 kcal/mol (**Figure 2E** and **Table 2**). IC docked in the binding site by interacting with various residues of STPK; Ala188(IC-O7—O-Ala, 2.83 Å), Gln187 (IC-O6—O-Gln, 2.86), Arg137 (IC-O2—NH1-Arg, 3.22 Å), Gly190 (IC-O14—O-Gly, 2.93 Å), and Arg161 (IC-O6—NH1-Arg, 2.68 Å) with a docking score of −4.74 kcal/mol (**Figure 2F** and **Table 2**). The ligand MRCT67127 was docked in the binding site where it showed few interactions with the surrounding residues, Arg189 (MRCT67127-N7—O-Arg, 3.04 Å). Arg137 (MRCT67127-N5—NH1-Arg, 2.85 Å), and Gln187 (MRCT67127-N1—NE-Gln, 3.04 Å), and it had a dock score of −4.25 kcal/mol (**Figure 2H** and **Table 2**). UA bound to the STPK showed a single hydrogen bond with the residue Arg137 (UA-O3—NH1-Arg, 2.94 Å) with a dock score of −2.65 kcal/mol (**Figure 2G** and **Table 2**). The docking studies showed a slightly higher binding of IC to STPK compared to the standard ligand MRCT67127.

**Table 2:** The dock scores and MM-GBSA binding energies for the ligands and reference compounds in complex with the DosR and STPK from *M. smegmatis* and *Mtb* all energies are in Kcal/mol with standard deviation in parenthesis.

Ligands	*M. smegmatis*				*M. tuberculosis*			
	DosR		STPK		DosR		STPK	
	Dock Score	MM-GBSA (ΔG_bind_)*	Dock Score	MM-GBSA (ΔG_bind_)	Dock Score	MM-GBSA (ΔG_bind_)	Dock Score	MM-GBSA (ΔG_bind_)
IC	-5.92	-52.96 (5.38)	-4.74	-43.41 (4.68)	-4.98	-31.76 (4.31)	-5.82	-24.30 (6.10)
UA	-3.55	-11.80 (5.89)	-2.65	-10.95 (4.77)	-2.35	-22.28 (7.33)	-3.61	-26.94 (4.09)
BA	-3.24	-30.66 (6.90)	-2.24	-24.84 (5.68)	-2.71	-17.13 (4.81)	-2.49	-33.86 (5.05)
HC104A	-4.27	-38.52 (2.87)	-4.25	-19.51 (3.47)	-3.97	-34.98 (6.11)	-5.11	-36.04 (7.94)
MRCT67127	-4.75	-34.50 (6.03)	-4.25	-29.43 (3.33)	-5.70	-33.45 (3.58)	-4.40	-50.83 (3.66)

*ΔG_bind_ = binding free energy (kcal/mol).

**Figure 3:**
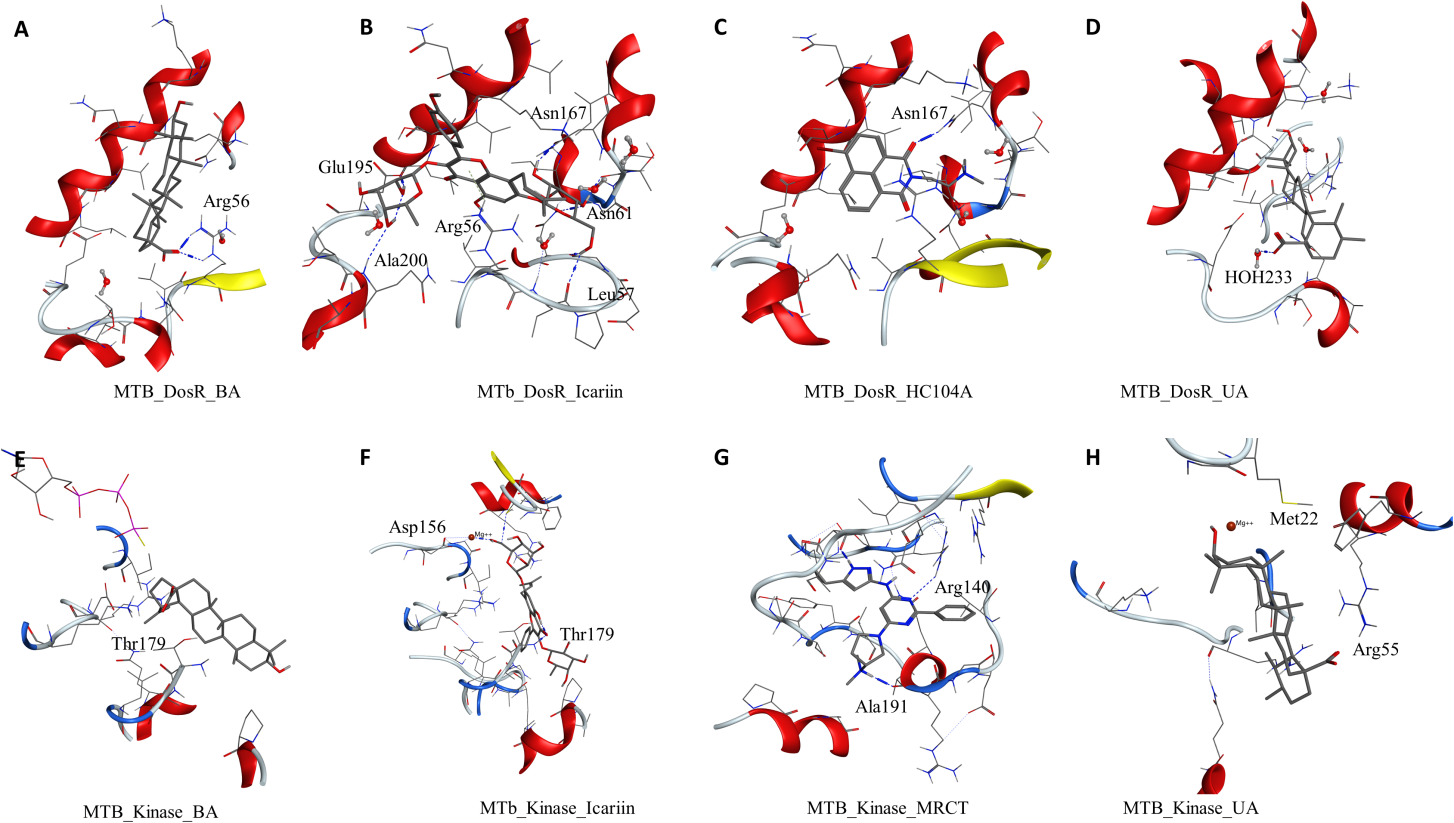
Molecular docking results for *M. tuberculosis* dormancy survival regulator (DosR) (PDB: 3C3W): **(A)** DosR–BA, **(B)** DosR–IC, **(C)** DosR–HC104A, and **(D)** DosR–UA. Molecular docking results for *M. tuberculosis* serine/threonine protein kinase (STPK) (PDB: 1MRU): **(E)** STPK–BA, **(F)** STPK–IC, **(G)** STPK–MRCT67127, and **(H)** STPK–UA.

To understand and compare the effect of the ligands on various important proteins and targets in the dormant state of the mycobacteria, we explored the *M. smegmatis* ICL, GMP synthase, LuxR, DosR, and STPK by docking experiments. DosR and STPK are reported to play a major role in the dormant state; hence, it was imperative to study these targets in *Mtb*. The crystal structure of DosR and STPK of *Mtb* are available in the PDB; hence, we explored these for the docking studies. The binding of ligand BA–DosR showed the formation of hydrogen bond and its interaction with the residue Arg56 (BA-O2—NH2-Arg, 2.70 Å) with a dock score of −2.71 kcal/mol (**Figure 3A** and **Table 2**). IC docked with the *Mtb* DosR and formed several hydrogen bond interactions owing to its free hydroxyl groups, Glu195 (IC-O13—OE2-Glu, 2.63 Å), Leu57 (IC-O9—O-Leu, 2.92 Å), Leu165 (IC-O8—O-Leu, 2.68 Å), Asn167 (IC-O6—OD1-Asn, 2.70 Å), and Asn61 (IC-O8—ND2-Asn, 3.12 Å), with a dock score of −4.98 kcal/mol (**Figure 3B** and **Table 2**). The ligand HC104A docked in the *Mtb* DosR with a dock score of −3.97 kcal/mol and formed a single hydrogen bond with the Asn167 (HC104A-O2—ND2-Asn, 2.86 Å) (**Figure 3C** and **Table 2**). The ligand UA did not form any hydrogen bond with the binding site residues of the *Mtb* DosR, and it showed a dock score of −2.35 kcal/mol (**Figure 3D** and **Table 2**). We also investigated the *Mtb* STPK to compare with the *M. smegmatis* STPK. The ligand BA did not form any interaction with the residues of the binding site of the *Mtb* STPK, for which the dock score was −2.49 kcal/mol (**Figure 3E** and **Table 2**). The ligand IC formed hydrogen bond interaction with the residues Thr179 (IC-O2—N-Thr, 3.07 Å), and it also coordinated with the magnesium ion present near the binding site near Asp156. IC showed a dock score of −5.82 kcal/mol (**Figure 3F** and **Table 2**). The ligand MRCT67127 docked in the binding site of *Mtb* STPK forming two hydrogen bond interactions with the residues Ala191 (MRCT67127-N2—O-Ala, 2.84 Å) and Arg140 (MRCT67127-N2—NH-Arg, 2.80 Å) with a dock score of −4.40 kcal/mol (**Figure 3G**). When UA was docked in the binding site of the *Mtb* STKP, it did not form any hydrogen bond interactions and showed a dock score of −3.61 kcal/mol (**Figure 3H**). These results from the docking of various ligands into the *Mtb* STPK suggest that IC binds better than MRCT67127 with regard to binding interactions with the residues of the binding site and the dock score. These observations raised some questions about the nature of ligand binding, stability of the ligand–receptor complex, and the binding energies of these complexes. To answer these questions, we performed molecular dynamics simulations (MDS) on all the complexes using explicit solvent model for 100 ns and used this trajectory to calculate the binding energies by MM-GBSA method (**Table 2**).

**Figure 4:**
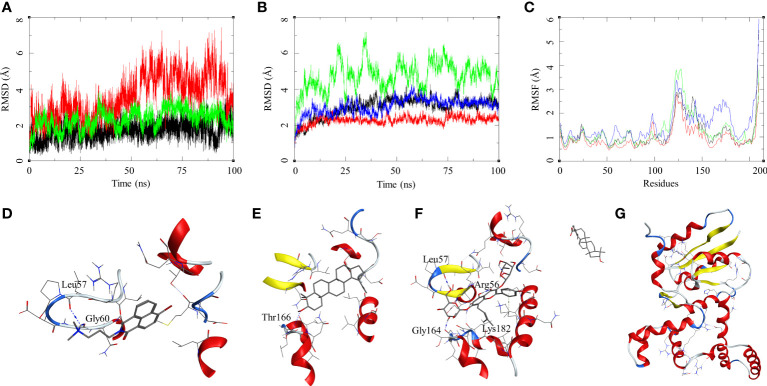
Molecular dynamics simulation results for *M. smegmatis* dormancy survival regulator (DosR) complexes: **(A)** ligand RMSD for HC104A (black), BA (red), and IC (green); **(B)** ligand-bound protein RMSD for HC104A (black), BA (red), IC (green), and UA (blue); **(C)** ligand-bound protein RMSF for HC104A (black), BA (red), IC (green), and UA (blue). Protein–ligand complex poses at the end of the 100-ns simulations, **(D)** DosR-HC104A, **(E)** DosR–BA, **(F)** DosR–IC, and **(G)** DosR–UA.

The ligand RMSD for compounds bound to the *M. smegmatis* DosR showed variable RMSDs. The ligand HC104A had low RMSD, which fluctuated between 1 and 2.5 Å throughout the duration of the simulation (**Figure 4A**), whereas IC showed a fluctuating trend with RMSD between 1 and 4 Å. The compound BA had a low RMSD between 1 and 4 Å, which rose to 2–7 Å for the last 60 ns; however, it returned to the lower RMSD state towards the end of the simulation. The ligand UA did not remain in the complex, as it broke away from the complex and ended up to the walls of the periodic box (**Figure 4G**). The RMSD of the receptor participating in the ligand–receptor complex was calculated, except for the IC-bound complex where the protein RMSD stayed below 3 Å throughout the simulation. The IC-bound protein showed fluctuations throughout the simulation; it kept between 2 and 6 Å, suggesting high flexibility of the complex due to the shape of the IC and high binding affinity (**Figure 4B** and **Table 2**). The RMSF was calculated to understand the effect of ligand binding. The ligands bound in the region surrounded by residues Arg56, Leu57, Gly60, Gly164, Thr166, Lys182, and other binding site residues. The RMSF in the region of 150–200 residues showed lower RMSF for ligand-bound complexes, whereas, in case of the UA-bound complex, a high RMSD in this region of the plot was observed (**Figure 4C**). The protein–ligand complexes were analyzed at the end of the simulations. HC104A bound to the *M. smegmatis* DosR retained its interaction with the Gly60 and formed a new hydrogen bond interaction with Leu57 (HC104A-N2—O-Leu, 3.11 Å) (**Figure 4D**). Ligand BA was found to remain in the binding site of the *M. smegmatis* DosR, but it lost its interaction with the binding site residues (**Figure 4E**). IC formed a new interaction with Gly164 (IC-O7—O-Gly, 2.95 Å) while retaining most of its original interactions during the MDS (**Figure 4F**). The binding energies of the *M. smegmatis* DosR-bound ligands were calculated by MM-GBSA, which suggested the highest binding energy for IC (ΔG_bind_ = −52.96 (5.38)) followed by HC104A (ΔG_bind_ = −38.52 (2.87) kcal/mol) and BA (−38.52 (2.87) kcal/mol). UA formed a very unstable complex with the *M. smegmatis* DosR, which is reflected by a low binding energy (ΔG_bind_ = −11.80 (5.89) kcal/mol). The molecular docking studies, MDS, and binding energies suggest for a stable complex between IC and *M. smegmatis* DosR.

**Figure 5:**
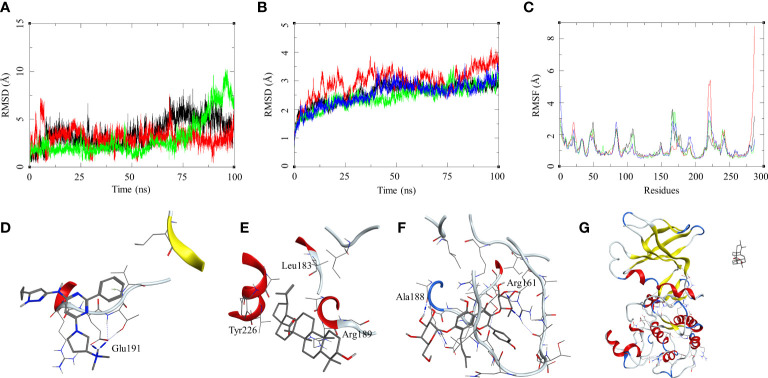
Molecular dynamics simulation results for *M. smegmatis* serine/threonine-protein kinase (STPK): **(A)** ligand RMSD for MRCT67127 (black), BA (red), and IC (green); **(B)** ligand-bound protein RMSD for MRCT67127 (black), BA (red), IC (green), and UA (blue); **(C)** ligand-bound protein RMSF for MRCT67127 (black), BA (red), IC (green), and UA (blue). Protein–ligand complex poses at the end of the 100-ns simulations, **(D)** STPK–MRCT67127, **(E)** STPK–BA, **(F)** STPK–IC, and **(G)** STPK–UA.

The ligand RMSD for those in complex with the *M. smegmatis* STPK showed a similar trend that we observed in an earlier case. Ligand MRCT67127 fluctuated between RMSD 1 and 7 Å; the RMSD for BA was found below 5 Å through the 100-ns MSD. Ligand IC showed a low fluctuation of <3 Å for the first 60 ns, after which it showed a gradual rise in the RMSD to 10 Å, and later, it reduced to 7.5 Å at the end of the MDS (**Figure 5B**). The ligand RMSD for UA was very high, as it leaves the complex right at the beginning of the MDS (**Figure 5G**). The protein RMSD for the complexes displayed a similar trend with an RMSD range between 1.5 and 4 Å, although the MDS was 100 ns. The protein RMSF for the complexes in the region of ligand binding show the effect of ligand binding. The binding site in the case of STPK lies in the region of residue numbers 160–200. The ligand-bound protein had a lower RMSF compared to the unbound one; the MRCT67127-, BA-, and IC-bound protein had lower fluctuations than the one bound to UA by approximately 1 Å (**Figure 5A–C**). The analysis of MDS trajectories towards the end of the simulations showed that the interaction profile of the ligands with several modifications happened during the MDS (**Figure 5D, E**). The ligand MRCT67127 lost its original interactions and formed new and more stable interactions with the STPK binding site residues. It formed hydrogen bond interactions with the Glu191 (MRCT67127-OE2—N7-Glu, 2.80 Å) and an arene–backbone nitrogen interaction *via* hydrogen with the Val193. The ligand BA did not retain any interaction with the binding site residues, but it remained in the bound state towards the end of the MDS. The ligand IC, on the other hand, retained almost all interactions that were found in the initial state of MDS except for the hydrogen bond with the Ala188 (**Figure 5F**). The ligand UA did not retain its interaction with the *M. smegmatis* STPK in the MDS (**Figure 5G**). The binding energies for the protein–ligand complex reflected the same trend with the *M. smegmatis* STPK-IC complex showing the highest binding energy (ΔG_bind_ = -43.41 (4.68) kcal/mol). The ligands MRCT67127 (ΔG_bind_ = −29.43 (3.33) kcal/mol) and BA (ΔG_bind_ = −24.84 (5.68) kcal/mol) were the second and third, respectively, whereas the ligand UA (ΔG_bind_ = −10.95 (4.77) kcal/mol) showed the lowest binding energies. These results suggest that IC was the best binding ligand for *M. smegmatis* STPK from this set of compounds.

The protein–ligand interactions between the natural products and standard compounds under investigation with the *M. smegmatis* targets, ICL, GMP synthase, LuxR, DosR, and STPK, exhibited the potential pattern of interaction, which suggest DosR and STPK to be the leading target proteins for these molecules. The molecular docking and MDS results along with the binding energy calculations favored these two receptors from *M. smegmatis* as top interacting partners. Taking the hint from these calculations and observations, we decided to investigate further for their selectivity in the case of *Mtb*. The rationale behind this study is to identify ligands that are active in the dormant stage of the mycobacteria. Honaker et al. (2009) and Sharma and Tyagi (2016) reported the importance of DosR activation in *Mtb* for the anaerobic survival, latent infection, and drug tolerance. Based on our *in silico* finding with MSM, we decided to proceed further with investigating the *Mtb* DosR and STPK, and fortunately, the crystal structures for both these *Mtb* targets are available in the PDB. The details about these targets are provided in the materials section (**Supplementary Table S2**). The docking studies, molecular dynamics simulations, and binding energy calculations were performed on all the natural products and the standard drugs under investigation.

**Figure 6:**
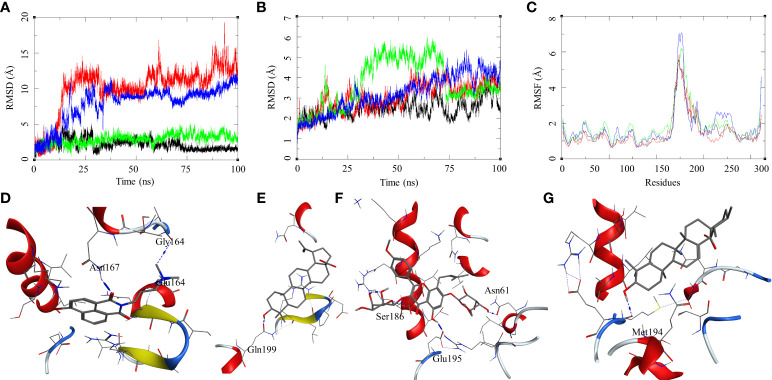
Molecular dynamics simulation results for *M. tuberculosis* dormancy survival regulator (DosR) (PDB: 3C3W): **(A)** ligand RMSD for HC104A (black), BA (red), IC (green), and UA (blue); **(B)** ligand-bound protein RMSD for HC104A (black), BA (red), IC (green), and UA (blue); **(C)** ligand-bound protein RMSF for HC104A (black), BA (Red), IC (green), and UA (blue). Protein–ligand complex poses at the end of the 100-ns simulations, **(D)** DosR–HC104A, **(E)** DosR–BA, **(F)** DosR–IC, and **(G)** DosR–UA.

The ligand RMSD for compounds bound to the *Mtb* DosR showed variable RMSDs. The ligand HC104A and IC showed a low ligand RMSD between 1 and 4 Å. The ligand BA showed a gradually rising RMSD over a period of 20 ns from 1 to 12.5 Å, after which it converged and stayed within a fluctuating range of 1–5 Å for the rest of the MDS. The ligand UA had a similar trajectory with a gradual rise in the RMSD from 1 to 10 Å over a period of 30 ns followed by a convergence between 1 and 5 Å for rest of the MDS (**Figure 6A**). The protein RMSDs for these complexes was almost similar except for the protein bound to IC. The protein RMSD fluctuated between 2 and 5 Å for HC104A, BA, and UA complexes. The IC-bound protein showed a rise in RMSD between 30 and 75 ns of the MDS. The rise was of approximately 3–s4 Å from its initial state; after 75 ns, the RMSD went steeply back to its original state (**Figure 6B**). This also reflected in the RMSF of the IC-bound protein, which had an overall higher RMSF, and this could be attributed to the size and higher conformational flexibility of the IC ligand (**Figure 6C**). Analysis of ligand binding interaction towards the end of the MDS was performed to understand the interaction of ligands with the binding site residues. HC104A formed new interactions during the MDS. Apart from its hydrogen bonding with the Asn167, it formed new bonds with the residues Gly164 (HC104A-N2—O-Gly, 3.94 Å) and Glu64 (HC104A-N2—OE2-Glu, 2.90 Å) (**Figure 6D**). The ligand BA stayed within the binding site; it lost bonding with the Arg56 but forms a new interaction with Gln199 (BA-O1—OE1-Gln, 2.58 Å) (**Figure 6E**). IC was successful in maintaining its interactions with the binding site residues during the MDS with some conformational changes (**Figure 6F**). The ligand UA did not show any interactions with the binding site residues during the initial stage of the MDS, but it formed a hydrogen bond with Met194 (UA-O1—O-Met, 2.58 Å) towards the end of the MDS (**Figure 6G**). The binding energies of these complexes were calculated over all the frames of the MDS of which the standard ligand HC104A showed the highest binding energy of ΔG_bind_ = −34.98 (6.11) followed by IC (ΔG_bind_ = −31.76 (4.31) kcal/mol), UA (ΔG_bind_ = −22.28 (7.33) kcal/mol), and BA (ΔG_bind_ = −17.13 (4.81) kcal/mol) (**Table 2**). These results were expected because ligand HC104A is a known *Mtb* DosR inhibitor (Zheng et al., 2020); this also strengthens our experimental results about the efficacy of the IC as an inhibitor of DosR in the mycobacteria.

**Figure 7:**
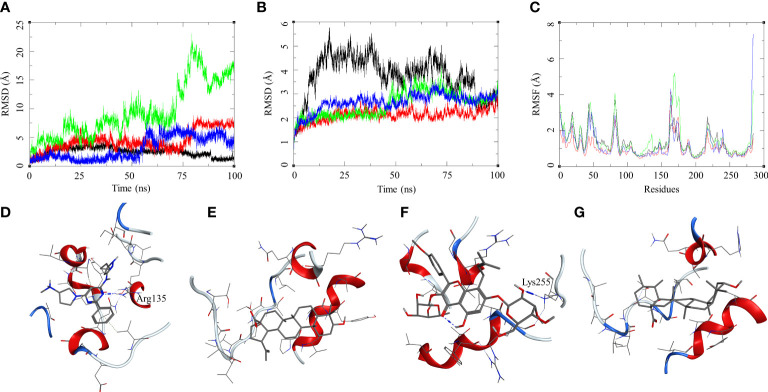
Molecular dynamics simulation results for *M. tuberculosis* serine/threonine-protein kinase (STPK) (PDB: 1MRU) complexes: **(A)** ligand RMSD for MRCT67127 (black), BA (red), IC (green), and UA (blue); **(B)** ligand-bound protein RMSD for MRCT67127 (black), BA (red), IC (green), and UA (blue); **(C)** ligand-bound protein RMSF for MRCT67127 (black), BA (red), IC (green), and UA (blue). Protein–ligand complex poses at the end of the 100-ns simulations, **(D)** STPK–MRCT67127, **(E)** STPK–BA, **(F)** STPK–IC, and **(G)** STPK–UA.

The ligand RMSD for compounds bound to the *Mtb* STPK showed variable RMSDs. The ligand RMSD for MRCT67127 remained low, between 1 and 5 Å, with very low fluctuations. However, BA showed a slightly higher fluctuation between 1 and 5 Å for approximately 75 ns of the MDS, after which there was a rise in the RMSD by approximately 5 Å till the end of the MDS (**Figure 7A**). In case of IC, there were high fluctuations throughout the MDS; it rose from 2.5 to 5 during first 20 ns and kept rising at an interval of 5 Å till the ligand showed very high deviation of approximately 20 Å. The visual inspection of the trajectory showed high conformational space exploration by IC. The ligand UA showed a stable RMSD initially, which was below 3 Å, but it gradually rose to approximately 6 Å around 60 ns and converged till the end of the MDS (**Figure 7A**). In case of the protein RMSD, the protein bound to MRCT67127 had high RMSD fluctuating between 1 and 5 Å throughout the MDS. The protein RMSD for the rest of the complexes was between 3 Å (**Figure 7B**). The protein RMSF for all the complexes showed a similar pattern for the ligand binding region of 150–200 residues, but in the case of the IC-bound protein, it was slightly higher by approximately 2 Å (**Figure 7C**). Analysis of ligand binding interaction towards the end of the MDS was performed to understand the interaction of ligands with the binding site residues. The ligand MRCT67127 retained its position in the binding site residues and formed a hydrogen bond interaction with the residue Arg135 (MRCT67127-N5—NH2-Arg, 2.80 Å), suggesting a stable complex that is supported by the ligand RMSD (**Figure 7D**). The ligand BA remained in the binding site of the *Mtb* STPK but did not participate in any interactions with the surrounding residues (**Figure 7E**). The ligand IC underwent large conformational change as evident from its ligand RMSD, towards the end of the MDS wherein it formed the hydrogen bond interaction with Lys255 (IC-O6—NZ-Lys, 2.92 Å) (**Figure 7F**). The ligand UA remained bound to the *Mtb* STPK through the MDS but did not form any hydrogen bond interactions (**Figure 7G**). The binding energies for these complexes was calculated over the MDS, the standard ligand MRCT67127 showed the highest binding energy of ΔG_bind_ = −50.83 (3.66) followed by BA (ΔG_bind_ = −33.86 (5.05) kcal/mol), UA (ΔG_bind_ = −26.94 (4.09) kcal/mol), and the IC (ΔG_bind_ = −24.30 (6.10) kcal/mol) (**Table 2**). These results were expected because ligand MRCT67127 is a known *Mtb* STPK inhibitor (Lougheed et al., 2011); this also strengthens our experimental results about the efficacy of the natural compounds as inhibitor of mycobacteria in its dormant state.

## Conclusion

We demonstrated the generation of dormant bacilli under stress environment, which showed loss of acid fastness, altered morphology, and tolerance to isoniazid and rifampicin. UA, BA, and IC effectively inhibited dormant population of *M. smegmatis.* For this study, an *in vitro* dormancy model for screening phytomolecules against a model strain of *M. smegmatis* was generated, and the activity was evaluated through hypoxic resazurin reduction assay.

*In silico* studies have provided deeper understanding about the possible interactions between the ligands and various targets investigated in this study. The molecular level interactions, binding modes of ligands with the binding site residues, and their behaviors in the solution phase helped in correlating the biological activities of the molecules on a structural basis. These investigations a part of this study have helped to determine important targets in the model strain of *M. smegmatis*, which have also been found in *M. tuberculosis.* DosR and STPK were found to be the targets common in both species that were more prone to these phytomolecules, which had promising inhibitory activity towards the dormant stage of *M. smegmatis*, and it could also be the case in *Mtb* as per the insights proved by the *in silico* studies.

## Future prospective

The current anti-TB therapy contain important drugs to kill the replicating form of bacilli, but they do not effectively work against drug-tolerant dormant population of bacilli. The identification of drugs that can eradicate phenotypically drug-tolerant dormant population of *Mtb* is important for developing effective regimens to shorten the treatment duration for TB.

There are further needs for studying the efficacy of these phytomolecules against the non-replicating form of *Mtb.* These targets can also be explored for target-based study in an *in vitro* model, which can provide clear results regarding the efficacy of these phytomolecules. These compounds might ultimately lead to new therapeutics or adjuvants, which can be used with first-line drugs to reduce the duration of lengthy anti-TB therapy after evaluation through different experimental approaches in the future.

## Data availability statement

The original contributions presented in the study are included in the article/**Supplementary Material**. Further inquiries can be directed to the corresponding author.

## Author contributions

SS and NS performed *in vitro* studies and microscopic analysis. RC performed modeling studies. VG designed the study and analyzed data. AK reviewed the manuscript. All authors contributed to the article and approved the submitted version.
