# Supplementary figures and images for: Targeting dormant phenotype acquired mycobacteria using natural products by exploring its important targets: In vitro and in silico studies
Source: Front Cell Infect Microbiol. 2023 Mar 24;13:1111997. doi: 10.3389/fcimb.2023.1111997 (PMC10080046; doi:10.3389/fcimb.2023.1111997)

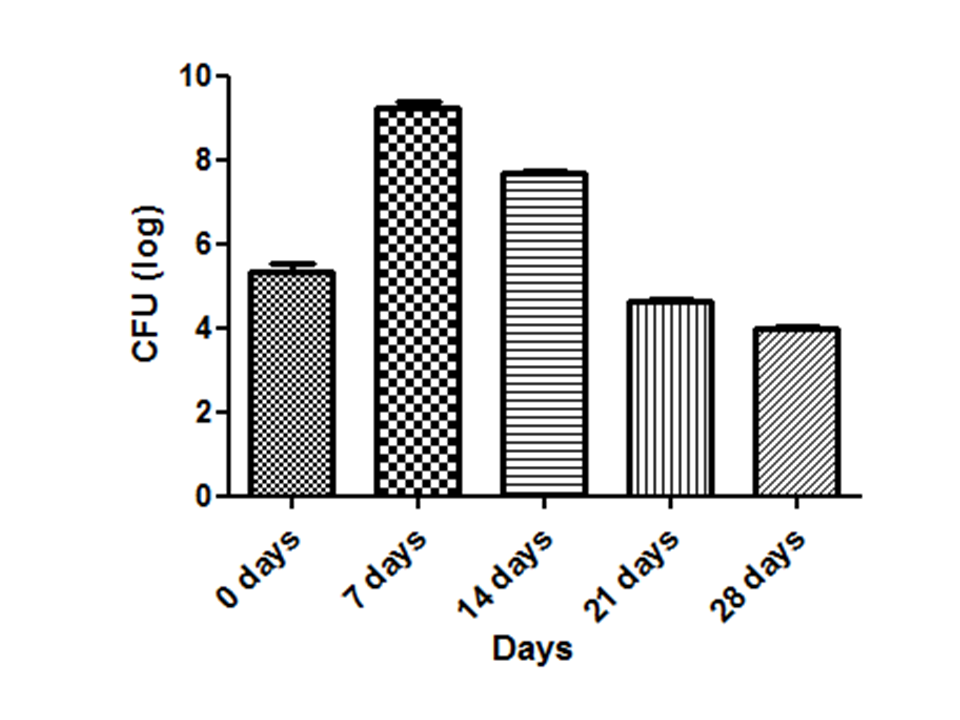

Supplement: Supplementary file 1 [file Image_1.tif]

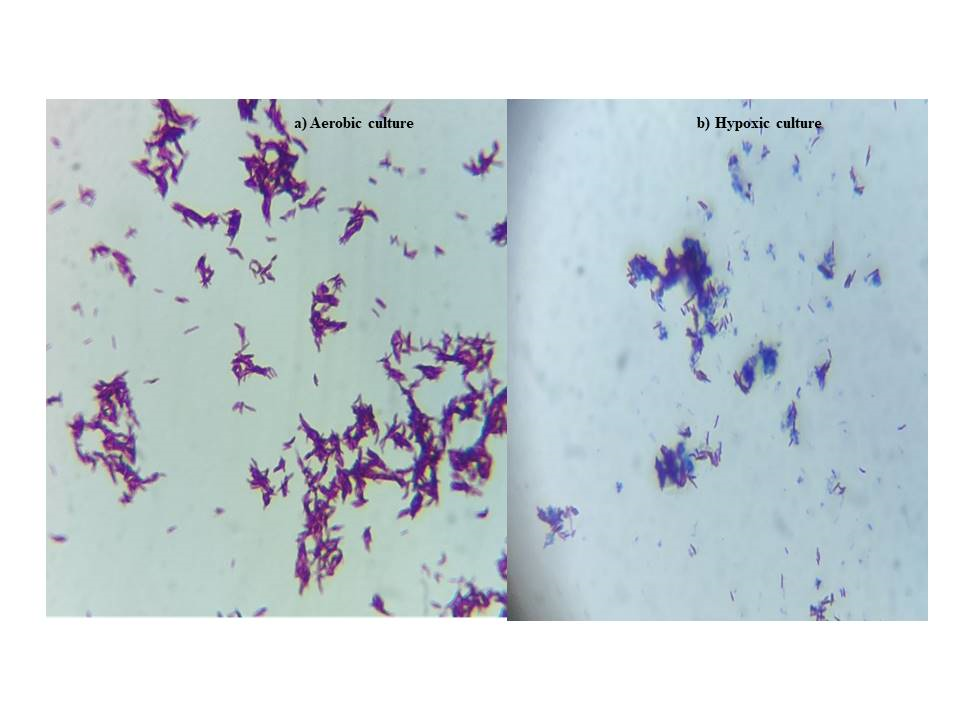

Supplement: Supplementary file 2 [file Image_2.tif]

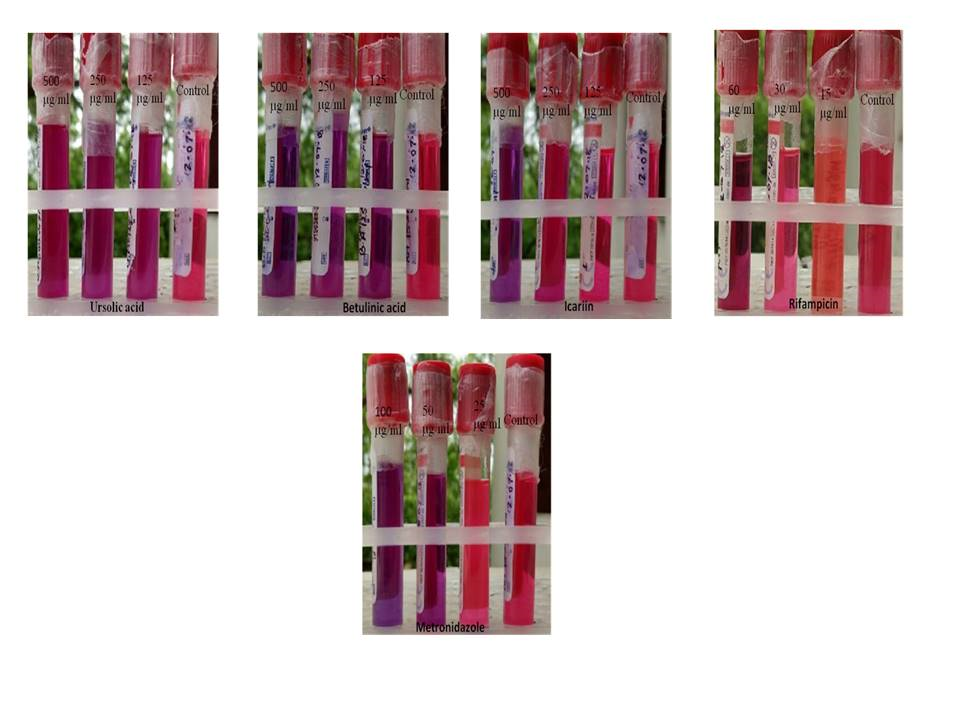

Supplement: Supplementary file 3 [file Image_3.tif]

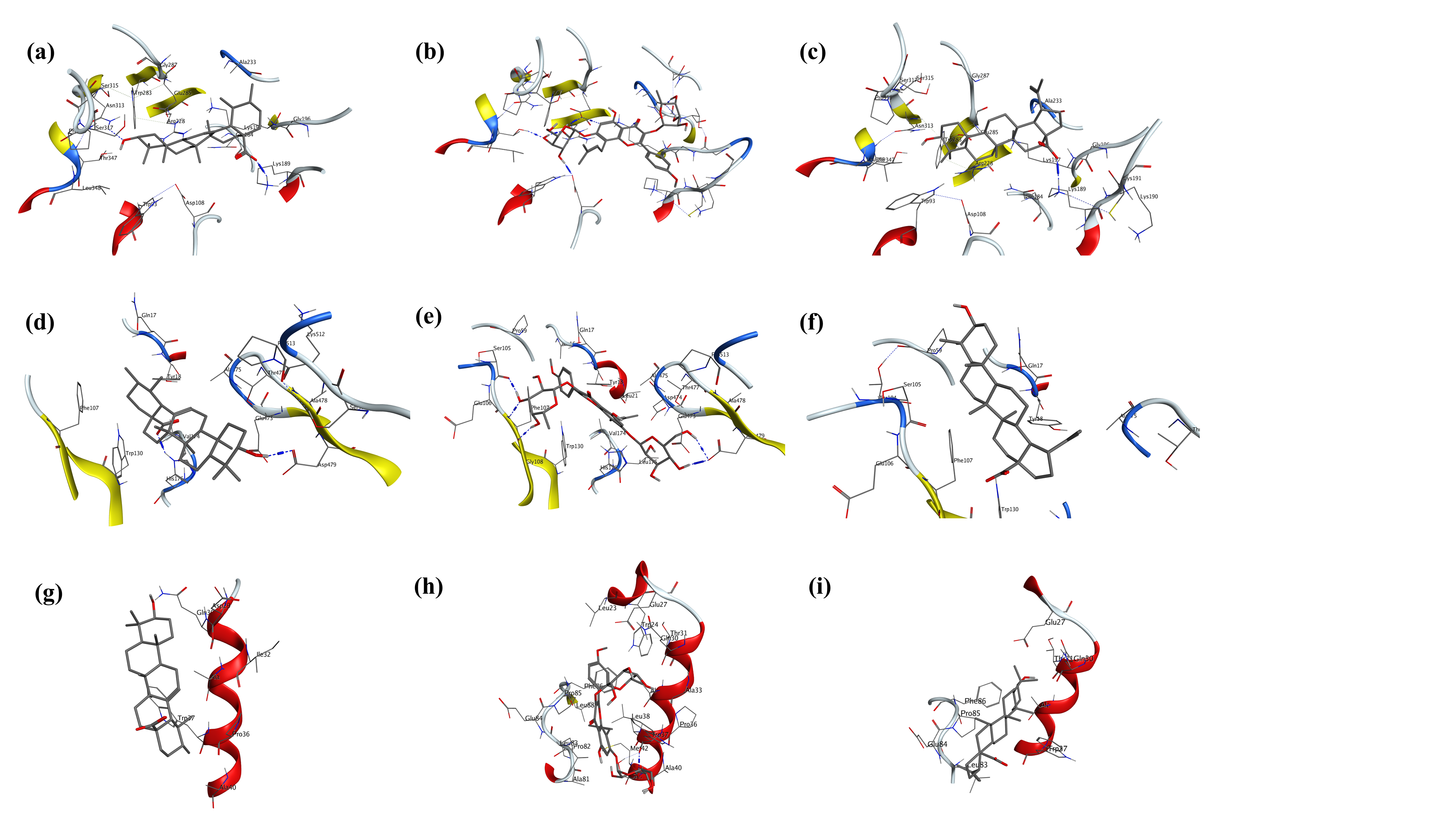

Supplement: Supplementary file 4 [file Image_4.tif]

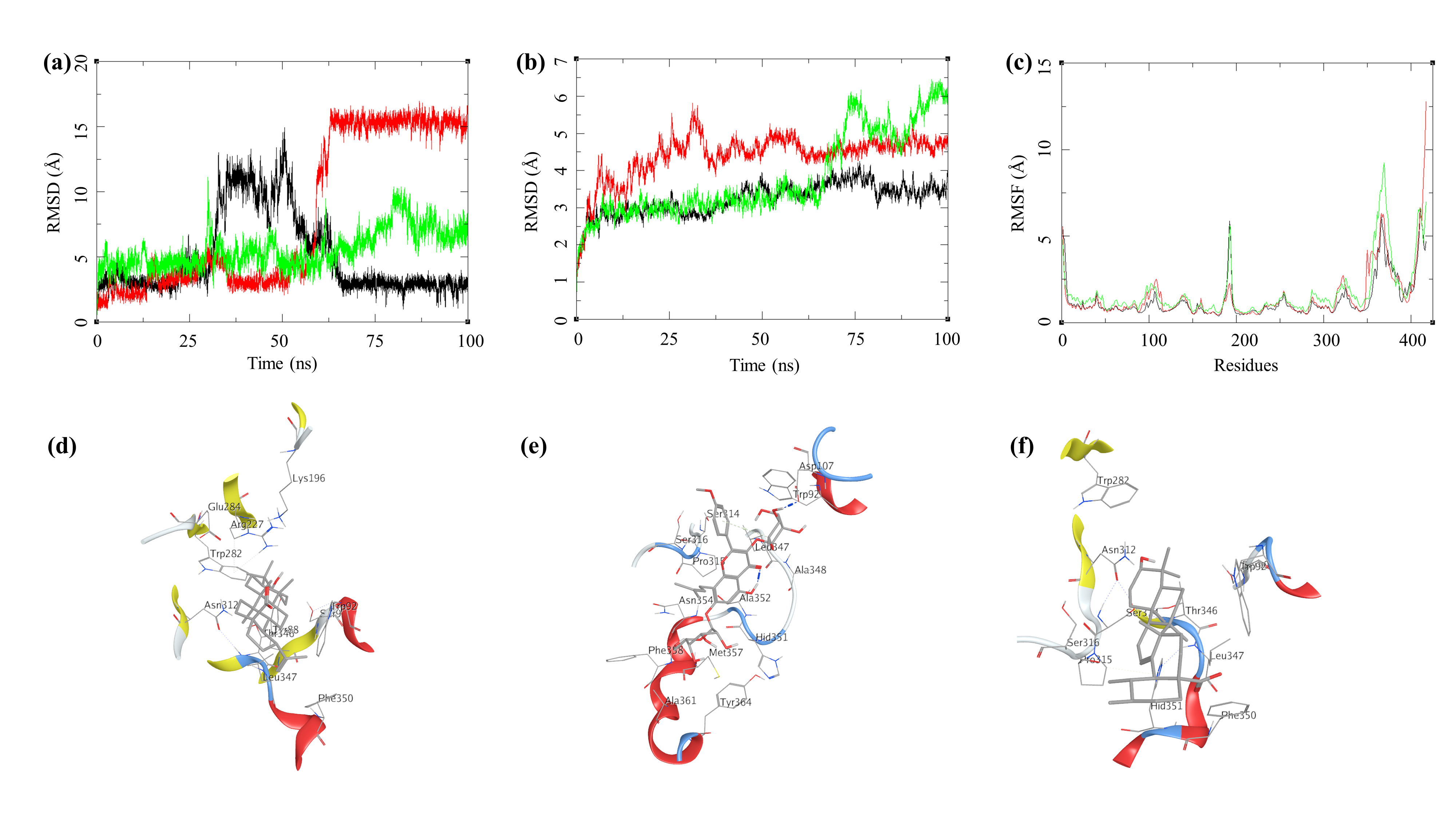

Supplement: Supplementary file 5 [file Image_5.tif]

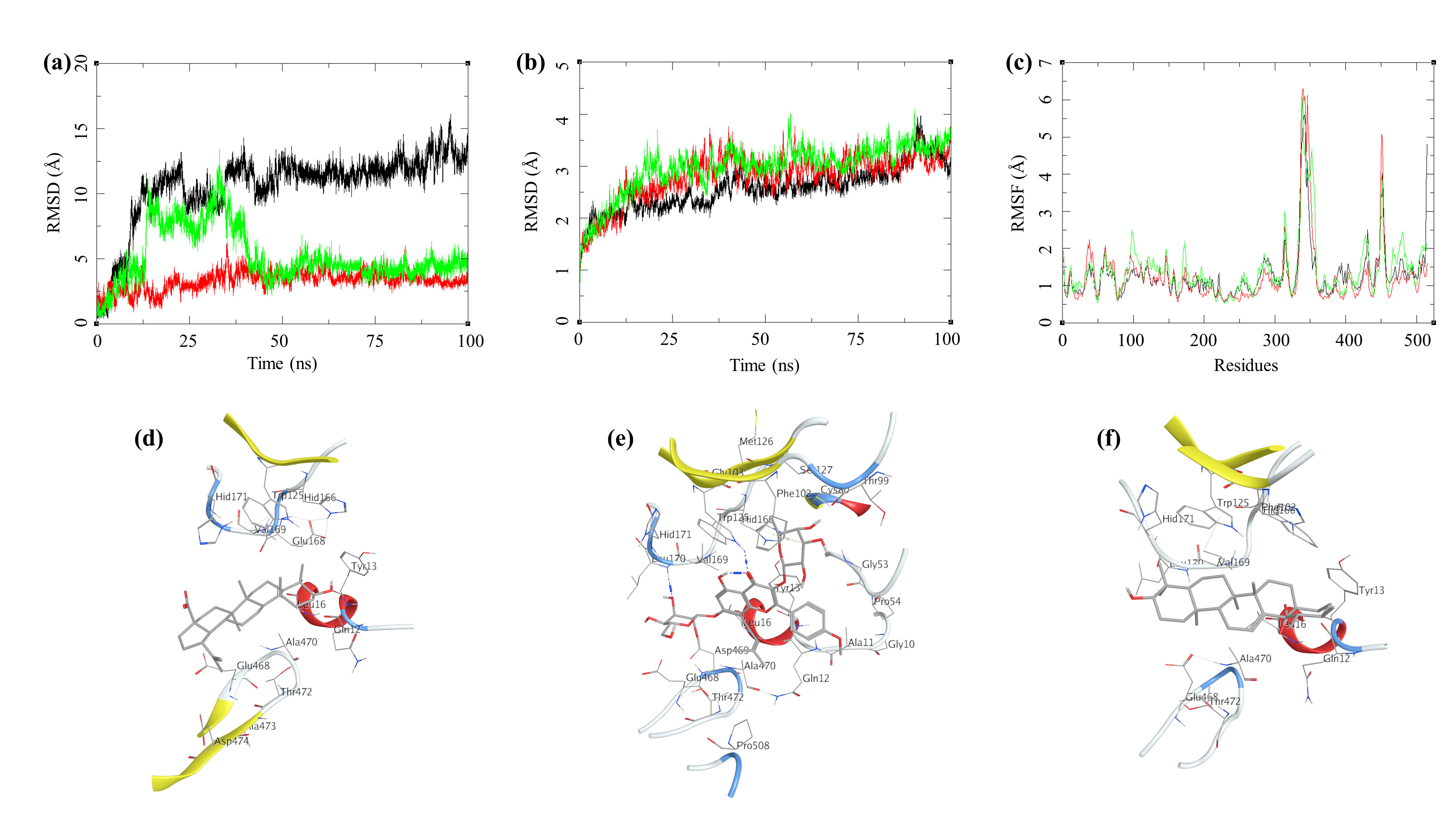

Supplement: Supplementary file 6 [file Image_6.tif]

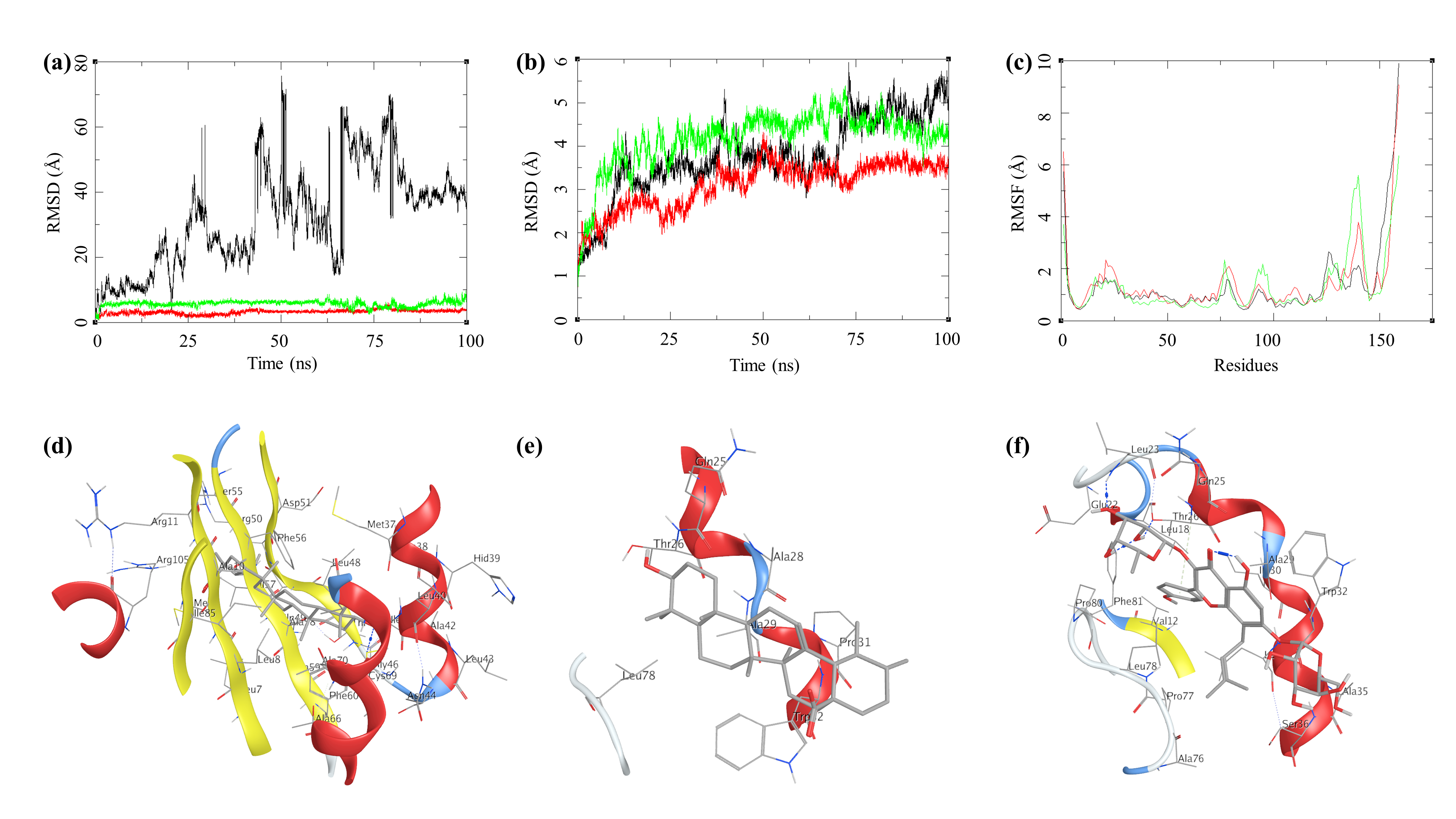

Supplement: Supplementary file 7 [file Image_7.tif]
