# Supplementary material for: Targeting dormant phenotype acquired mycobacteria using natural products by exploring its important targets: In vitro and in silico studies
Source: Front Cell Infect Microbiol. 2023 Mar 24;13:1111997. doi: 10.3389/fcimb.2023.1111997 (PMC10080046; doi:10.3389/fcimb.2023.1111997)
Supplement: Supplementary file 8 [file Table_1.docx]

**Targeting dormant phenotype acquired mycobacteria using natural products by exploring its important targets: *In vitro* and *in silico* studies**

Shweta Sharma^1┼^, Rupesh Chikhale^2,3┼^ Nivedita Shinde^1^, A. M. Khan^4^, Vivek Kumar Gupta^1*^

^1^Department of Biochemistry, ICMR-National JALMA Institute for Leprosy and Other Mycobacterial Diseases, Agra-282004, India

^2^Division of Pharmacy and Optometry, University of Manchester, Oxford Road, Manchester, United Kingdom

^3^Department of Pharmaceutical & Biological Chemistry, School of Pharmacy, University College London, 29-39 Brunswick Square, London, WC1 N 1AX UK

^4^ Division of Clinical and Implementation Research, ICMR-National JALMA Institute for Leprosy and Other Mycobacterial Diseases, Agra-282004, India

| **S. No** | **Ingredients** | Amount (L) |
| --- | --- | --- |
| 1 | L-Asparagine | 4.0gm |
| 2 | di-Pottasium hydrogen phosphate | 0.5gm |
| 3 | Ferric ammonium citrate | 0.05gm |
| 4 | Glycerol | 60ml |
| 5 | Magnesium sulphate heptahydrate | 0.7gm |
| 6 | Tri-Sodium citrate | 2.0gm |
| 7 | Tween-80 | 0.05% |
| 8 | Zinc sulphate | 0.01gm |

**Supplementary Material**

Supplementary Table 1. Modified Sauton’s medium composition (pH 6.0)

Supplementary Table 2. Details from the Uniprot database, Homology modelling and validation of *M. smegmatis* target receptors.

| **Sr. No.** | **Target receptor name** | **Uniprot details** | **RC plot** | **MolProbity Score** | **Qmean score** |
| --- | --- | --- | --- | --- | --- |
| ***M. smegmatis* target receptors** | | | | | |
| 1 | Isocitrate lyase | A0QQX6 · A0QQX6_MYCS2  *Mycolicibacterium smegmatis* (strain ATCC 700084 / mc(2)155) (*Mycobacterium smegmatis*)  Isocitrate lyase | 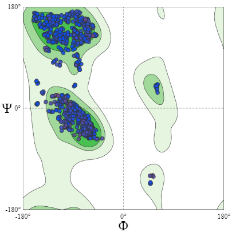 | 0.64 | 0.91 |
| 2 | GMP Synthase | A0QSV0 · A0QSV0_MYCS2  *Mycolicibacterium smegmatis* (strain ATCC 700084 / mc(2)155) (*Mycobacterium smegmatis*)  GMP synthase [glutamine-hydrolyzing] | 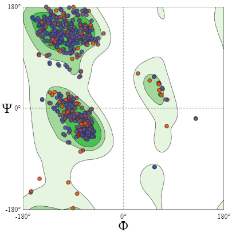 | 1.64 | 0.74 |
| 3 | LuxR | A0QPW8 · A0QPW8_MYCS2  Mycolicibacterium smegmatis (strain ATCC 700084 / mc(2)155) (*Mycobacterium smegmatis*)  Transcriptional regulator, LuxR family protein | 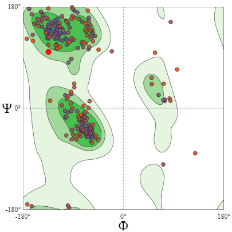 | 2.12 | 0.62 |
| 4 | DosR | A0R2V2 · A0R2V2_MYCS2  *Mycolicibacterium smegmatis* (strain ATCC 700084 / mc(2)155) (*Mycobacterium smegmatis*)  Two component transcriptional regulatory protein devr | 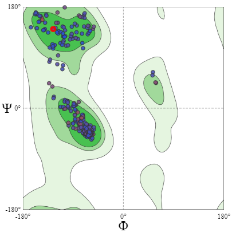 | 1.62 | 0.81 |
| 5 | STPK | A0QNG1 · PKNB_MYCS2  *Mycolicibacterium smegmatis* (strain ATCC 700084 / mc(2)155) (*Mycobacterium smegmatis*)  Serine/threonine-protein kinase PknB | 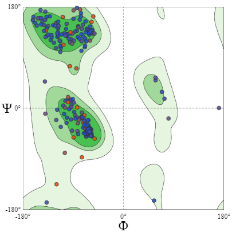 | 0.95 | 0.82 |
| ***M. tuberculosis* target receptors** | | | | | |
| 1 | DosR | P9WMF9 · DEVR_MYCTU  *Mycobacterium tuberculosis* (strain ATCC 25618 / H37Rv)  DNA-binding transcriptional activator DevR/DosR | 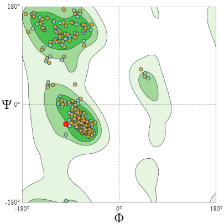 | 2.08 | 0.84 |
| 2 | STPK | P9WI81 · PKNB_MYCTU  *Mycobacterium tuberculosis* (strain ATCC 25618 / H37Rv)  Intracellular Ser/Thr protein kinase domain of *Mycobacterium tuberculosis* PknB | 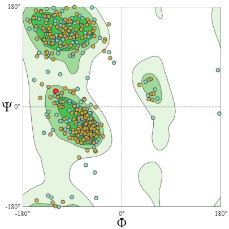 | 3.37 | -3.44 |

Supplementary Table 3. The dock scores (Kcal/mol) and MM-GBSA binding energies (Kcal/mol) for the ligands in complex with the Isocitrate lyase, GMP synthase and LuxR from *M. smegmatis.*

| **Ligands** | ***M. smegmatis*** | | | | | |
| --- | --- | --- | --- | --- | --- | --- |
|  | **Isocitrate lyase** | | **GMP Synthase** | | **LuxR** | |
|  | **Dock Score** | **MM-GBSA** | **Dock Score** | **MM-GBSA** | **Dock Score** | **MM-GBSA** |
| Icarin | -5.95 | -32.90 (4.90) | -5.29 | -36.39 (5.41) | -4.41 | -45.42 (5.15) |
| Ursolic acid | -3.54 | -21.09 (3.79) | -2.72 | -23.25 (5.24) | -2.03 | -23.18 (3.99) |
| Betulinic acid | -2.85 | -21.10 (4.84) | -1.89 | -28.88 (6.00) | -0.98 | -11.48 (5.82) |
| HC104A | -5.29 | -- | -5.08 | -- | -5.96 | -- |
| MRCT67127 | -4.05 | -- | -4.79 | -- | -7.57 | -- |

Supplementary Figure 1: Bar diagram showing growth of *M. smegmatis* under hypoxic environment at different time intervals

Supplementary Figure 2. Cultures of *M. smegmatis* after Z -N Staining

Supplementary Figure 3. HyRRA of *M. smegmatis* using different phytomolecules in vacutainer tubes (blue color: bacterial growth inhibition; pink color: bacterial growth)

Supplementary Figure 4. Molecular docking results for *M. smegmatis*: (a) *M. smegmatis* Isocitrate lyase- Betulinic acid (BA), (b) *M. smegmatis* Isocitrate lyase – Icariin, (c) *M. smegmatis* Isocitrate lyase – Ursolic acid (UA), (d) *M. smegmatis* GMP synthase – UA, (e) *M. smegmatis* GMP synthase – Icariin, (f) *M. smegmatis* GMP synthase – BA, (g) *M. smegmatis* LuxR – BA, (h) *M. smegmatis* LuxR – Icariin, (i) *M. smegmatis* LuxR – UA.

Supplementary Figure 5: Molecular dynamics simulation results for *M. smegmatis* Isocitrate lyase complexes: (a) Ligand RMSD for BA (Black), Icariin (Red), and UA (Green); (b) Ligand bound protein RMSD for BA (Black), Icariin (Red), and UA (Green); (c) Ligand bound protein RMSF for BA (Black), Icariin (Red), and UA (Green). Protein-ligand complex poses at the end of the 100ns simulations, (d) Isocitrate lyase -BA, (e) Isocitrate lyase -Icariin, and (f) Isocitrate lyase -UA.

Supplementary Figure 6: Molecular dynamics simulation results for *M. smegmatis* GMP synthase complexes: (a) Ligand RMSD for BA (Black), Icariin (Red), and UA (Green); (b) Ligand bound protein RMSD for BA (Black), Icariin (Red), and UA (Green); (c) Ligand bound protein RMSF for BA (Black), Icariin (Red), and UA (Green). Protein-ligand complex poses at the end of the 100ns simulations, (d) GMP-BA, (e) GMP-Icariin, and (f) GMP-UA.

SI-Figure 7: Molecular dynamics simulation results for *M. smegmatis* LuxR complexes: (a) Ligand RMSD for BA (Black), Icariin (Red), and UA (Green); (b) Ligand bound protein RMSD for BA (Black), Icariin (Red), and UA (Green); (c) Ligand bound protein RMSF for BA (Black), Icariin (Red), and UA (Green). Protein-ligand complex poses at the end of the 100ns simulations, (d) LuxR -BA, (e) LuxR -Icariin, and (f) LuxR -UA.
